# Are We Ill Because We Age?

**DOI:** 10.3389/fphys.2019.01508

**Published:** 2019-12-18

**Authors:** Tamas Fulop, Anis Larbi, Abdelouahed Khalil, Alan A. Cohen, Jacek M. Witkowski

**Affiliations:** ^1^Geriatrics Division, Department of Medicine, Research Center on Aging, University of Sherbrooke, Sherbrooke, QC, Canada; ^2^Singapore Immunology Network (SIgN), Biopolis, Agency for Science Technology and Research (A*STAR), Singapore, Singapore; ^3^Department of Microbiology and Immunology, Yong Loo Lin School of Medicine, University of Singapore, Singapore, Singapore; ^4^Department of Biology, Faculty of Sciences, University of Tunis El Manar, Tunis, Tunisia; ^5^Department of Family Medicine, Research Center on Aging, University of Sherbrooke, Sherbrooke, QC, Canada; ^6^Department of Pathophysiology, Medical University of Gdansk, Gdansk, Poland

**Keywords:** aging, age related diseases, aging as a disease, functional healthspan, anti-aging medicine, geroscience, adaptation, optimization of aging

## Abstract

Growing elderly populations, sometimes referred to as gray (or silver) tsunami, are an increasingly serious health and socioeconomic concern for modern societies. Science has made tremendous progress in the understanding of aging itself, which has helped medicine to extend life expectancies. With the increase of the life expectancy, the incidence of chronic age-related diseases (ARDs) has also increased. A new approach trying to solve this problem is the concept of geroscience. This concept implies that the aging process itself is the common cause of all ARDs. The corollary and consequence of such thinking is that we can and should treat aging itself as a disease. How to translate this into the medical practice is a big challenge, but if we consider aging as a disease the problem is solved. However, as there is no common definition of what aging is, what its causes are, why it occurs, and what should be the target(s) for interventions, it is impossible to conclude that aging is a disease. On the contrary, aging should be strongly considered not to be a disease and as such should not be treated; nonetheless, aging is likely amenable to optimization of changes/adaptations at an individual level to achieve a better functional healthspan.

## Introduction

There is no doubt our societies are aging. This has consequences for health, social, and familial care in any modern society. Thus, a response from the society to the question of how we will care for all these elderly persons is more and more urgently needed. It should be mentioned that this is not always approached only in positive ways, but many different negative opinions are also circulating, culminating in so-called “ageism” or partial exclusion of elderly from society. Furthermore, there is also a fear of what is called the “gray tsunami” or “silver tsunami” (Bartels and Naslund, 2013; Mitchell, 2014; Gregory, 2015; Barishansky, 2016; Bluethmann et al., 2016; Masselam, 2017; Berntsen et al., 2019; Rotman, 2019).

So, whether an aging society will overwhelmingly use societal and health resources is the most important question that gerontologists and geriatricians should answer. One response which can be evoked is that many specialists either in the field of gerontology or geriatrics are arguing that aging as such is a disease and so it can be treated or prevented *per se* (Bulterijs et al., 2015; Faragher, 2015; Gems, 2015; Zhavoronkov and Bhullar, 2015). Furthermore, this idea is coupled to the new approach to chronic diseases called geroscience, which states that as aging is the major risk factor for most chronic diseases, thus we should be able to decrease the occurrence of age-related chronic diseases (ARDs) by influencing or ideally preventing aging by any means (Sierra, 2016a,b; Sonntag and Ungvari, 2016; Yabluchanskiy et al., 2018). The earliest translation of this concept is the introduction of the frailty concept (Rockwood et al., 2000; Fried et al., 2001) trying to conceptualize aging as a treatable medical condition (Rodriguez-Mañas and Fried, 2015; Rockwood, 2016; Walston et al., 2019). In this review, we will discuss whether the only response to this new complex societal and medical challenge is to consider aging as a disease for better answering to the needs of the aging society, or whether other avenues should be explored. Therefore, we will also consider what we know on whether aging is treatable or curable, and how this interacts with the question of whether it is a disease. Ultimately, we will explore how this might change the regulatory frameworks for research and therapeutics in ways that could impact human health.

## What is Aging?

Understanding what is aging is very important for the understanding whether aging is a disease or not; consequently, we will approach the definition of aging from this perspective. We should acknowledge that there are many concepts and theories (more than 300) to explain aging (Rose et al., 2012; Cannon, 2015; Lipsky and King, 2015; da Costa et al., 2016). This multitude generates many ways of thinking, which may even be completely contradictory. However, it seems that some may be more inclusive than others, e.g., the evolutionary theories or the free radical theory of aging (Chandrasekaran et al., 2017; Reichard, 2017). Recently, the physiological dysregulation concept tried to approach better the understanding of the process of aging (Cohen et al., 2013). Nevertheless, none of these 300+ theories totally capture this very complex and multifactorial phenomenon. The concept of aging may be broadly conceptualized as the by-product of the passage of time. This would define aging as a natural, ever-progressing “deterioration” of physiological functions; an increased susceptibility to certain diseases; and an intrinsic, age-related process of loss of viability and increase in vulnerability leading ultimately to death (Strehler and Mildvan, 1960; Birren and Zarit, 1985; Strehler, 1985). However, aging may also be defined more positively as the result of a time-dependent adaptation which ultimately becomes maladapted (dysregulated), no longer obeying the principle of hormesis and leading to self-elimination (Calabrese, 2018). This concept strongly suggests that aging is random and purposeless.

Of course, there are many types of aging such as the physiological, biological, molecular, functional, or even social. Just as life is hierarchically organized from molecules to cells to tissues, organs, systems, organisms, and populations, aging can also occur at multiple organizational levels, with consequences for the others. Here we will mostly discuss biological aging; however, physiological aging will be also considered. One of the most integrative definitions proposed so far states that biological aging [sometimes called “senescing,” especially in relation to biological (cellular) phenomenon of “senescence”] is the process of change in the organism which over time decreases the probability of survival and reduces the physiological capacity for self-regulation, repair, and adaptation to environmental demands (Schroots and Birren, 1988). This definition integrates all important aspects defining biological aging, namely time, changes, decrease of reserves, dysregulation, and irreversibility of its ending with death. The definition supports the division of the aging process into primary aging, which is postulated to reflect an intrinsic, presumably genetically determined limit on cellular (and organismal) longevity (accounting for the relatively constant maximum lifespan observed in almost all animal species studied) and in secondary aging, due to the accumulated effects of environmental insults, disease, and stress (explaining most of the variability between individuals’ aging trajectories within the species) (Anstey et al., 1993). It should be strongly stressed that primary and secondary aging can most likely influence each other *via* a positive feedback loop. Together aging is considered time-dependent and complex, occurring at various levels of the organism, and can be characterized as universal, progressive, inevitable, and irreversible, though also to some extent modulable owing to the marked individuality of the process (Libertini, 2015; Weiss, 2018; Michel et al., 2019).

All these aspects were recently summarized as the “nine hallmarks of aging,” including the intercellular communication, genomic instability, telomere attrition, epigenetic alterations, loss of proteostasis, deregulated nutrient sensing, mitochondrial dysfunction, cellular senescence, and stem cell exhaustion (López-Otín et al., 2013). These hallmarks (or the similar framework of pillars, Kennedy et al., 2014) covers quite well the current knowledge of cellular and molecular mechanisms of aging. However, research in the field is advancing quickly, and there is good reason to suspect that these lists will change in the coming years, as new theories/mechanisms emerge, and others are discarded (as has recently happened for oxidative stress in a broad sense) (Hekimi et al., 2011). Interestingly, Hekimi et al. in contrast to the widely accepted theory on free radicals’ participation in aging process suggested that the increased production of free radicals is an adaptation to aging and is therefore beneficial (Wang and Hekimi, 2015). The hallmarks of aging are not the only one which change with the advent of new knowledge. For example, the hallmarks of cancer were recently redefined to include a much broader list than that presented in the original publication (Hanahan and Weinberg, 2011). These are examples of the constant progress of our knowledge which shapes also the definition of aging. Furthermore, the authors acknowledge explicitly that all these described mechanisms are tightly interconnected and consequently are useful ways to summarize many related mechanisms.

Thus, a thorough understanding of aging implies an integrative, complex systems framework where lower level mechanisms can have direct impacts (e.g., mutations causing cancer or cellular senescence), or indirect impacts *via* higher level processes (e.g., impacts of inflammation on atherosclerosis). This framework can help explain the diversity of aging patterns across the tree of life, with both “public” (universal) and “private” (species-oriented) mechanisms.

## Why do we Age?

This is a crucial question, however not easy to answer. There can be many responses depending on what definition of aging we adopt and the perspective we consider (physiological, evolutionary, etc.) (Kirkwood, 2017; Flatt and Partridge, 2018). If we consider the definition which we found the most applicable to the biology of aging, we could answer that time-dependent exhaustion at all levels of the aging body renders it unable to sufficiently sustain the physiological/molecular functions of the organism which consequently collapses. Such understanding implies that aging is an irreversible process, but does not exclude the possibility that it is amenable to modulations. However, this amenability is not equal to “treatability” and thus would not imply that aging is a disease.

A next, broader view to conceptualize why we age would include an evolutionary perspective (Le Bourg, 2014; Reichard, 2017). Classical evolutionary theories rely on the declining force of natural selection at older ages. From a population perspective, random (age-independent) mortality will reduce the probability of surviving at older ages, as the resources invested earlier in life will be more effective at increasing fitness. This principle, in different ways, underlies the mutation accumulation, antagonistic pleiotropy, and disposable soma theories of aging (Williams and Day, 2003; Kowald and Kirkwood, 2015). Nonetheless, recent findings suggest that a wide array of species from across the tree of life do not age at all (there is no increase in mortality with accumulating times of their lives) (Schaible et al., 2015); these findings imply that we do not yet have a sufficient answer for the question why *H. sapiens* and many other species age. Nonetheless, the comparative data are clear in implying that each species has an inherent aging rate (or lack thereof), and that individual variation in aging rate within a species is small relative to interspecies differences.

## What is a Disease and the Nature of the So-Called “Age-Related Diseases”?

The definition of disease is problematic from an epistemological perspective (Scully, 2004; Doust et al., 2017). It can be argued that no two individuals undergo exactly the same pathogenesis, so defining diseases is an exercise in grouping similar entities together. But at what point are two pathological entities sufficiently different to merit separate names? Is cancer a single disease, or many related diseases? We can consider either the similar manifestations of various causes such as in syndromes (e.g., Parkinsonism, Metabolic syndrome) or the etiology with differential clinical manifestations such as in a disease state (e.g., Parkinson disease, Diabetes mellitus type 2). In a very simple way, we can perhaps define an illness/disease as « a state » where the optimal physiological functioning of the body systems, cells, and/or the mind are perverted to a pathophysiological process causing symptoms of a pathology. Thus, it would be these symptoms that together define the disease. The occurrence of a disease is the combination of the susceptibility of the host, the conducive environment, and finally the insult. In the case of communicable diseases, the aggressor is generally a pathogen (usually virus, bacteria, or fungus) which is propagating from person to person, while in non-communicable diseases it is determined by environmental hazards, life habits, and/or genetics which do not spread from person to person (Prüss-Ustün et al., 2019). Thus again, various diseases manifest themselves more or less specifically by objectively evidenced signs and symptoms (Poh et al., 2017). Considering these characteristics, it is clear that the causes and pathomechanisms can be different depending on the nature of the disease: caused by acute or chronic exposure to pathogens, by improperly executed physiological processes such as inflammation or auto-immunity, by genetic, biochemical, or environmental problems or failure in normal functioning/adaptability of an organ or organ system (dysregulation), ultimately becoming chronic after the initial aggression has been eliminated (Bury, 1982).

Most of the diseases affecting humans are chronic. Chronic diseases according to the WHO have many determinants which extend from the underlying socioeconomic determinants *via* common modifiable and nonmodifiable risk factors to intermediate risk factors such as high blood pressure, high blood sugar, or high lipids (The World Health Report, 2002; WHO Global Report, 2005). All these factors converge and to various extents determine the appearance of the most common chronic diseases which affect humans, such as cardiovascular diseases, type 2 diabetes, cancers, chronic respiratory diseases, or neurodegenerative diseases. The most widespread belief is that most of these diseases occur in and affect older subjects. If the global occurrence of these diseases is closely examined, half of the subjects suffering from them are under the age of 70 (WHO Global Report, 2005). If the causes of these diseases are considered closely, this is not surprising, as among the most prevalent, intricate, and intertwined causes, age as a nonmodifiable risk factor is only one and far from being the most determinant (Fülöp et al., 2019). Accordingly, it may be considered that age is an important but not a determinant risk factor for these diseases; however, when the individual is aging, accumulated changes in the organism such as the physiological dysregulation of numerous systems of the body permit the clinical manifestation of long-lasting, underlying, detrimental pathological processes (Franceschi et al., 2018; Fulop et al., 2018). Additionally, there is no disease that occurs inevitably with aging; thus, while aging is a risk factor, it is neither a necessary nor sufficient cause for chronic diseases (Rothman et al., 2008).

We will illustrate the above-mentioned pathophysiological development of chronic diseases by three clinical examples.

First, we will consider the case of cancer. This is a disease which develops throughout life (Anisimov, 2009). The original carcinogenic insult occurs most probably at a younger age, as we need some “driver mutations” to occur sequentially in order to promote neoplastic transformation (Busque et al., 2018; Sweet-Cordero and Biegel, 2019). This may or may not develop further into clinically manifesting malignancy. For the latter to occur, there is a need for the concerted actions of pro-oncogenic stimulations progressing from this initial insult through its promotion to the malignancy accompanied (or rather made possible) by the waning host resistance. This is the reason why the common thinking is that cancer is a “disease of older subjects.” The notion comes from the superficial evaluation of the clinical/epidemiological data. However, it is of note that after 90 years of age the incidence is decreasing, and many centenarians are exempt from cancer (Pavlidis et al., 2012). Together, cancer may be considered a disease starting in the young or middle age but manifesting later with aging.

The second group of ailments that we would consider are the cardiovascular diseases. They also start early in life as an inflammatory process called atherosclerosis (Ross, 1999). During autopsies on young soldiers dead in the Korean War, the signs of atherosclerotic lesions in the arteries have been discovered (Webber et al., 2012). It is of note that it progresses at different speeds in each individual, and even appears absent in some populations (Gurven et al., 2009). Thus, many adult subjects may suffer at various points of their life from the clinical manifestations of atherosclerosis which may increase with age. This strongly suggests that as we age, the clinical appearance of cardiovascular diseases may increase to some extent revealing the underlying lifelong atherosclerotic process due to the physiological changes in the organism. Once again, the underlying pathology occurs from the early ages, but the clinical manifestation may have an age-enriched clinical appearance.

Finally, the third example concerns the neurodegenerative diseases, such as spontaneous Alzheimer’s disease (AD). The insults, whatever they may be (including oxidative, metabolic, or infectious), occur decades before the clinical manifestation of the disease (Festoff et al., 2016; Le Page et al., 2018). Individual susceptibility and changes will determine whether these insults will result in clinically manifest AD. A good support for this notion is the presence of morphological features called the AD plaques even in the brains of cognitively normal elderly subjects (Duan et al., 2017). Thus, once more the insult occurs earlier in life, while the clinically manifest disease appears only much later, usually after the age of 60.

These examples throw doubt on the aging-caused nature of these diseases, typically considered as age-related, and even more that they could be prevented by treating the underlying aging process (as their “roots,” i.e., founding pathomechanisms, are present at earlier ages). Furthermore, most of the chronic disease burden today appears to be conditions that are rare or absent in hunter-gatherers, horticulturalists, etc. Nonetheless, contemporary hunter-gatherers do age, and the symptoms of aging appear to be similar to what is observed in the healthiest older individuals in modern society: wrinkled skin and decreased speed, strength, and lower endurance likely related to sarcopenia (Fuellen et al., 2019). The diseases mentioned above are thus not manifestations of aging, but of the cumulative effects of decades of modern lifestyles and environmental exposures.

In summary, a disease is a perturbation in the homeostasis of the organism presenting itself by specific (e.g., hemiplegia in case of stroke) and non-specific symptoms (e.g., unconsciousness in case of stroke). Chronic diseases have various pathogenic pathways, likely starting early in life; however, the only common pathway at the end may be inflammation. While inflammation may increase the risk of multiple chronic diseases, even this is not universal: centenarians have very high levels of inflammatory markers, and the same is true for some hunter-gatherers (Salvioli et al., 2009; Rubino et al., 2019). Accordingly, chronic diseases are not only the attribute of older subjects, but also of a considerable proportion of younger sufferers. Age may be at most a nonmodifiable risk factor playing a role in the clinical appearance of these chronic diseases, but by no means their cause. Considering the above-mentioned examples, a fundamental logical error underlying the geroscience approach may be the confusion of time-dependence with age-dependence. Of course, this does not imply that specific interventions into aging hallmarks can never have benefits related to chronic disease. For example, it is possible and even likely that targeted senolytics (drugs removing senescent cells or preventing their formation) approaches will have benefits for specific diseases in specific population; nonetheless (Kirkland et al., 2017), even geroscience experts caution that broad application of senolytics could likely have substantial negative health consequences (Blagosklonny, 2018a; Attaallah et al., 2019).

## What may be the Reason That Aging is Considered as a Disease?

The answer to this question is not easy, even if intuitively some would say “yes, aging is a disease and as such may be cured” (Caplan, 2005; Bulterijs et al., 2015; Zhavoronkov and Bhullar, 2015; Stambler, 2017) while others will say “not at all, aging is not a disease although it may be modulated” (Rattan, 2014). Even if this distinction seems trivial, it has many consequences for science and translational research. Why did this concept emerge and what is the consequence? The notion of aging as disease comes mainly from animal studies, as some manipulations have proven to be effective in increasing lifespan and decreasing chronic diseases, the most successful being the caloric restriction (Colman et al., 2009; Anisimov, 2015; Johnson and Kaeberlein, 2016; Mattson et al., 2017; Kraig et al., 2018). This raised interest in a similar approach in humans, aiming at a medicalization of aging (Justice et al., 2018a; Blagosklonny, 2018b). From a social point of view, there is a need for a response how to cope with the “silver tsunami” economically, socially, and psychologically (Bartels and Naslund, 2013; Mitchell, 2014; Gregory, 2015; Barishansky, 2016; Bluethmann et al., 2016; Masselam, 2017; Berntsen et al., 2019; Rotman, 2019). Finally, fighting against specific chronic diseases is not sufficiently rapid and efficient, and moreover it is very costly (Yabluchanskiy et al., 2018; Larsen, 2019), leading to the idea that, by fighting aging, medicine could prevent or at least retard the appearance of all the chronic diseases simultaneously.

It is clear that the simple passage of time – *chronological* aging – is relentless and unstoppable. Recently, some experts studying aging say it is time for a fresh look at this *biological* process – and recognize aging as a condition that can be manipulated, delayed, and possibly treated, unlike time itself (Gems, 2015). They argue that institutional and ideological barriers are standing in the way to recognize aging as a disease, and a major one is the longstanding traditional view that aging is not a disease, but a natural, benign process that should not be interfered with. “Because aging is not viewed as a disease, the whole process of bringing drugs to market can’t be applied to drugs that treat aging. This creates a disincentive to pharmaceutical companies to develop drugs to treat it” and “If aging is seen as a disease, it changes how we respond to it: it becomes the duty of doctors to treat it.” (Kelland, 2010; Gems, 2015). Furthermore, according to Dillin, “there is now a ‘groundswell’ of specialists in aging who are lobbying the world’s biggest drug regulator, the U.S. Food and Drug Administration, to consider redefining aging as a disease in its own right” (Kelland, 2010; Riera and Dillin, 2015). What is the *primum movens* behind this willingness to consider aging as a disease? If we would be only trivial, we would say money, but we would like to hope that there are other considerations behind this lobbying. There are probably also more hidden considerations from the societal aspect which says that if we have longer healthspan (Kaeberlein, 2018), it would mean longer productive lifespan and less need for pensions, though this could only be true if the proportion of life spent pre-retirement increased. Consequently, some authors, such as de Grey, have even compared delay in researching aging as equivalent to murder (De Grey and Rae, 2007).

Therefore, there is a strong movement in gerontology to consider aging as a disease, which would facilitate the search for drugs to intervene in the process of aging (in the “aging disease”) and eventually extend the healthspan and concomitantly the lifespan (Hansen and Kennedy, 2016). This way of thinking is a corollary to anti-aging medicine which aims to rejuvenate the aged subjects (Gems, 2014; Longo et al., 2015; de Magalhães et al., 2017; Espeland et al., 2017; Melo Pereira et al., 2019). Accordingly, the pointed interventions in the process of aging would prevent the damage leading to pathology. It was partly demonstrated in animal models concerning some aspects of body maintenance, but certainly not with the intention to cure aging as would be required if aging would be the disease that they state. Alternatively, repairing the aging-related damages as the anti-aging medicine proposes should lead to a decrease in pathologies. Thus, from the “aging as a disease” point of view, the one thing that science is missing is to consider and so investigate aging as the common cause/ mechanism for all of chronic diseases of the elderly, rather than being interested in investigating and curing the specific ARDs. This way of considering aging raises the fundamental question of whether modifying aging would really affect the risk for the occurrence of these ARDs. Would the proposed interventions decreasing the burden of ARDs concomitantly slow the aging process? Alternatively, would they slow aging because they prevent diseases that accelerate aging?

Furthermore, there is a fundamental lack of definition of how geroscience is considering healthspan, which is a composite, multidimensional state (Moskalev et al., 2016; Sierra, 2016a,b; Blagosklonny, 2018a). Already the definition of health took decades to be agreed on and even then it is changing with the progress of medicine, so the definition of healthspan should be similarly considered. Is it defined by the absence of diseases, by the quality of life (which is personal and subjective), or ultimately by the number of pills that an old person is taking? Most of the elderly do not even know or feel that they are ill. The following example from the clinical experience of one of us can help visualize this. The case is of the 99-year-old woman who had fallen at home and has been hospitalized. She did not take any medication and has not seen the doctor for the last 10 years. Could we then assume that she was in good “subjective health” so her healthspan could extend until her fall? However, while she was in the hospital, she was diagnosed with several chronic diseases including arrhythmia, hypertension, aortic stenosis, mild COPD, and type 2 diabetes and started to be treated for them. She felt awful because she learned from doctors how seriously ill she was. Finally, treatment was implemented, she got better and she returned home suffering from several ARDs. Of course, this is not a typical example; however, there are likely millions of such cases. It was shown that self-reported health was more positive if the seniors were not limited in their functionality even if they have been diagnosed with chronic diseases (Maddox, 1999; Yoshimitsu et al., 2017). This suggests that, in contrast to what is often stated, the many different changes related to the normal aging process *per se* diminish functionality only very slightly, even if they lead to ARD (Lipsky and King, 2015). In the spirit of “aging is a disease” all of these persons should be treated to attain a hypothetical healthspan. Thus, it would be of paramount importance to find a universal definition of healthspan, which may apply as an ultimate and universal goal for treating aging as if it was a disease.

And this is the problem: advocates of “aging as disease” seem to believe that they can find means (drugs or procedures) to (partially or altogether) free an old person from the symptoms of aging that had already occurred, and in consequence, from ARDs. On the other hand, those like us who do not consider aging as a disease, but a natural (but modifiable) process in life, would advocate for modifying the functioning of organism at any relevant level BEFORE any symptoms of aging and ARDs occur, and thus prolonging healthspan, ARD-free and overall lifespan.

## How Geroscience and Frailty are Integrated in the Notion That Aging is a Disease and What are the Translational Implications

Geroscience (already mentioned earlier a few times) is an interdisciplinary field that aims to understand the relationship between aging and age-related diseases (Sierra, 2016a,b). Because aging is considered the major risk factor for most non-genetic chronic diseases, an understanding of the role of aging in the onset of these diseases should open up new avenues for disease prevention and cures. This term also describes an interdisciplinary approach to the biology of aging. However, because a factor (aging) is a risk factor (for ARDs), it does not become itself a disease. By this logic, sedentarity which is a major risk factor for many metabolic and cardiovascular diseases should be also considered per se as a disease, but obviously it is not.

There are presently nine recognized hallmarks for aging, as mentioned above (López-Otín et al., 2013). Interventions are proposed aiming at targeting one or several of these aging hallmarks in hopes of having an impact on ARDs. Considered therapies include stem cell therapy, immune/inflammation modulators, senolytics to eliminate senescent cells, telomerase activation, epigenetic modulatory drugs, activation of chaperones and proteolytic pathways, dietary interventions by modulating/inhibiting mTOR and the Insulin/IGF-1 Signaling (IIS) pathways as well as activating the AMPK and the sirtuins, and finally modulating the mitochondria metabolism and genesis (Longo et al., 2015; Vaiserman et al., 2016; Fedichev, 2018; Mitchell et al., 2018; Mitteldorf, 2018; Justice et al., 2018b; Sharma and Padwad, 2019). The question arises as to whether we can conceive a holistic approach to the mentioned interventions to target most of the pillars of aging, thereby resulting in a decrease in ARDs.

These are very useful interventions as proved in animal models; however, they all target some specific aspects of the aging process. None of them has a universal modulatory effect except perhaps some type of nutritional interventions (e.g., caloric restriction, intermittent fasting, and calorie restriction mimetics). However, even these interventions may not target all hallmarks, such as proteostasis (Witkowski et al., 2018; Konopka et al., 2019). Thus, the proposed interventions do not tackle the high complexity of aging. Consequently, considering the complex nature of aging, if we intervene in one aspect, we do not know what the effects on the others will be. Likely, a possibly further deleterious disequilibrium would be created if we consider the physiological dysregulation concept of aging. Thus, we are back to the fundamental questions what is aging and why we age. Until we can answer these questions with confidence, the uncertainty about the role of all these intertwined processes in the complexity of aging could render the proposed interventions dangerous in the short or long term. For example, it is possible that senolytic interventions will succeed in slowing aging at several levels but will simultaneously increase cancer risk by suppressing Senescence Associated Secretory Phenotype (SASP). SASP may have both detrimental and beneficial effects on cancer, and the relative balance would need to be studied in detail (Blagosklonny, 2018a; Zhang et al., 2019). However, given the normal structure of clinical trials, it might be easy to observe short-term benefits in aging biomarkers and other health indices, and harder to observe changes in cancer risk that might happen over longer timeframes.

For example, the newly conceptualized changes in the immune system in connection with the idea of inflammaging (subclinical state of increased inflammatory readiness, manifested by elevated levels of proinflammatory cytokines) made many of our beliefs related to immunosenescence obsolete (Franceschi et al., 2018; Fulop et al., 2018; Pawelec, 2018). A more recent interpretation of these changes considers age-related immune changes as immunoadaptation necessary for longevity, as shown in case of the semi-supercentenarians (Arai et al., 2015) and in the case of the most modern vaccination practices devised for seniors (Lal et al., 2015). However, when this immunoadaptation is dysregulated, then the aged individuals become subject to late manifestation of age-related diseases. Thus, until we really know what the exact role of a process considered “age-related” is, any intervention to “modulate” or to “cure” it may be very harmful instead of being beneficial or resulting in increasing healthspan. This distinction between adaptation and pathology during aging is crucial and underemphasized.

The same can be said concerning the largely used and studied phenotype called frailty (Fried et al., 2001; Rockwood, 2016). There are numerous definitions of frailty as well as measurements suggesting that we really do not know what it is, as with the various measurements we are capturing just one aspect of it (Theou et al., 2013; Malmstrom et al., 2014; Mijnarends et al., 2015). It is clear that if we refer to the basic definition of frailty as the loss of the body’s reserves leading to a decreased resilience and consequently to adverse events, we are just giving the physiological definition of aging (Fulop et al., 2010; Khan et al., 2019). Together this would signify a failure of the homeodynamic maintenance of system functions (Rattan, 2015). Alternatively, we can conceive of multiple levels of failsafe mechanisms, with most clinical manifestations occurring when the last failsafe mechanism disappears. This goes back to the idea of redundancy as discussed by the Gavrilovs (Gavrilov and Gavrilova, 2004). Thus, ultimately frailty seems no more than just a certain state resultant from the biological aging process which is different in each individual; this is clearly underlined as the prevalence of clinical frailty varies from 7 to 60% in diferent aging populations. This notion could be very useful if it would be used as a definition of biological aging rather than used as a medicalization of aging which should be at any cost treated (Palliyaguru et al., 2019).

These considerations suggest that geroscience – considering aging as the root of all ARDs and as such amenable to any interventions – is not a useful approach to understand aging. This also further suggests that aging should not be considered as a disease and interventions aiming at modulating/postponing aging should not be designed as if it was a disease. Therefore, we need to clearly define why aging is not a disease and suggest other ways to approach the question of aging and age-related diseases.

## Why Aging is not a Disease?

In the optic of geroscience, if aging becomes a treatable disease/process, it will be the duty of medical doctors to treat it. However, not everything which seems to be aging is aging. Over the history of gerontology and geriatrics, many processes previously thought to be part of aging are now considered not to be age-related, but an overlaying pathology. One of the best examples is anemia, which for decades was considered as a solid attribute of aging but now is considered related to various pathologies and not to aging itself (Halawi et al., 2017). So, an older individual who does not have relevant underlying pathomechanisms would not have anemia even at 100 years of age or more. The same applies to hypertension, to sarcopenia, to kidney failure, and to cognitive impairment (Khan et al., 2017; Sobamowo and Prabhakar, 2017).

So again, what distinguishes aging from a disease conceptually? First, the extent of aging is systemic and complex while that of a disease is mostly limited. Aging is an inevitable, universal process (concerning all humans living long enough) while most diseases are associated with individuals’ susceptibilities/vulnerabilities, and most of them, even chronic, are preventable. The most important cause of aging is time, while diseases usually have specific known causes. In other words, aging is irreversible and progressive while diseases are reversible and discontinuous. Finally, and most importantly, aging may be modulable but not treatable, while diseases are ultimately treatable even if we do not know presently how, which is only a question of progress of science. So many essential differences clearly speak against the notion that aging is “just another” disease.

Moreover, we should state that every older adult is different, while most of the people suffering from the same disease are fundamentally identical with respect to the disease. In other words, aging process is much more heterogeneous than any disease would ever be (Li et al., 2016). This contention should lead, as we will describe later, to a different approach to the concept of aging and (if any) to interventions. This also indicates that the notion of individual aging signifies individual health status and perception which would define the functionality of the aged subject. Therefore, “one size fits all” does not work for aging. Aging is an adaptation, thus to “cure” it may cause more harm than good. Considering these arguments, we can state that aging s not a disease.

## Is There a Place for Anti-aging Medicine?

The corollary of geroscience in the medical field is anti-aging medicine, which tries to “cure” aging by rejuvenating older individuals (Gammack and Morley, 2004; Rattan, 2004; Bartke, 2008; Balistreri, 2018). This is made by the modulation of the extrinsic attributes of aging such as wrinkled skin (e.g., by hyaluronic acid or botox injections) (Draelos, 2008; Taub and Pham, 2018) or the intrinsic attributes such as sarcopenia (by stem cell treatment) (Chhetri et al., 2018).

These treatments may have some beneficial effects in the short run but certainly are not causal treatments (Gammack and Morley, 2004). Indeed, they may apparently help to relieve specific aspects of aging but will not change the healthspan or the global functionality of the aging organism. Thus, these interventions do not take the complexity of aging into account. Presently, without solid scientific proof, there is no place in the medical arsenal for what we call anti-aging medicine, except for some trivial cosmetic interventions.

To extend the discussion, we should ask how we would know if an anti-aging therapy really could slow aging. The problem is that most of our definitions are circular or impractical. At the most macro level, we might ask whether it extends lifespan or life expectancy. However, based on this definition, seatbelts, obstetrical care, and childhood vaccinations are miraculous anti-aging interventions. Some laboratory interventions to slow aging may be highly dependent on the environment, and thus the extent to which they are slowing aging is debatable. Alternatively, we might ask if we reduce the incidence or burden of ARDs with anti-aging interventions. However, as noted above, it is possible we could do this by counteracting negative aspects of modern lifestyle (e.g., obesity, sedentarity), without affecting aging *per se*, and conversely that we might find interventions that slow aspects of aging without having much impact on ARDs. Lastly, we might ask whether anti-aging interventions have impacts on metrics of biological aging. If these metrics are specific metrics of the processes being treated, the reasoning becomes circular. For example, we could not prove that senolytics affect aging simply because they reduce the number of senescent cells. Higher level indicators of biological age, such as homeostatic dysregulation indices or the epigenetic clock, are slightly more promising metrics (Belsky et al., 2018). However, even here there is a problem: these various indices are only poorly correlated with each other and are themselves based on various theories about what aging is. For example, if senolytics lower (rewind) the epigenetic clock, is this simply because the epigenetic profiles of senescent cells are different, and we have removed these cells from the mix? Or was there really an impact on aging in the remaining cells? Thus, there is a fundamental conceptual challenge in defining aging that translates into an equivalent challenge in defining whether an anti-aging therapy is successful. This logical problem in turn propagates into the identification of pillars and hallmarks: the third and most crucial criterion for hallmarks of aging (though not always met in the published list) is that “its experimental amelioration should retard the normal aging process and hence increase healthy lifespan.” One example is metformin treatment. It may be that metformin, on average, reduces the burden of many chronic diseases (ARDs) and even improves quality of life. But does this mean it slows aging? Or if it does slow aging, does it only slow aging because it prevents diseases that accelerate aging? There are almost certainly human populations (e.g., hunter-gatherers, athletes) that would not benefit from metformin. It would not slow their aging. So, does it slow aging, or inhibit some of the negative consequences of modern lifestyles in a general way? Are these the same things? Are these negative consequences part of aging for us now or permanently? For these reasons, it is problematic to define the normal aging process and to measure whether it is retarded, and equally difficult to define healthy lifespan in ways that avoid the seatbelt problem mentioned above. Together, to answer the title question of this subchapter, at this stage of our knowledge there is no place in medicine for anti-aging medicine understood as treating symptoms of aging when aging has already happened. However, there might be a place for interventions/modulations that would delay the occurrence of aging, when applied early in life, before any time-dependent processes had accumulated and aging symptoms show up.

## How Alternatively Aging may be Conceptualized as not Being a Disease

Does all this signify that there is nothing which can be done for improving an aging individual’s health and functionality? Does it matter for older individuals being considered “ill” by virtue of their age? What would an aged person like for the end of his/her life? What is important for them? Does it really matter for them whether aging is considered a disease or not? These questions are rarely asked from the elderly themselves, with scientists thinking they know better what the elderly need.

**Figure 1:**
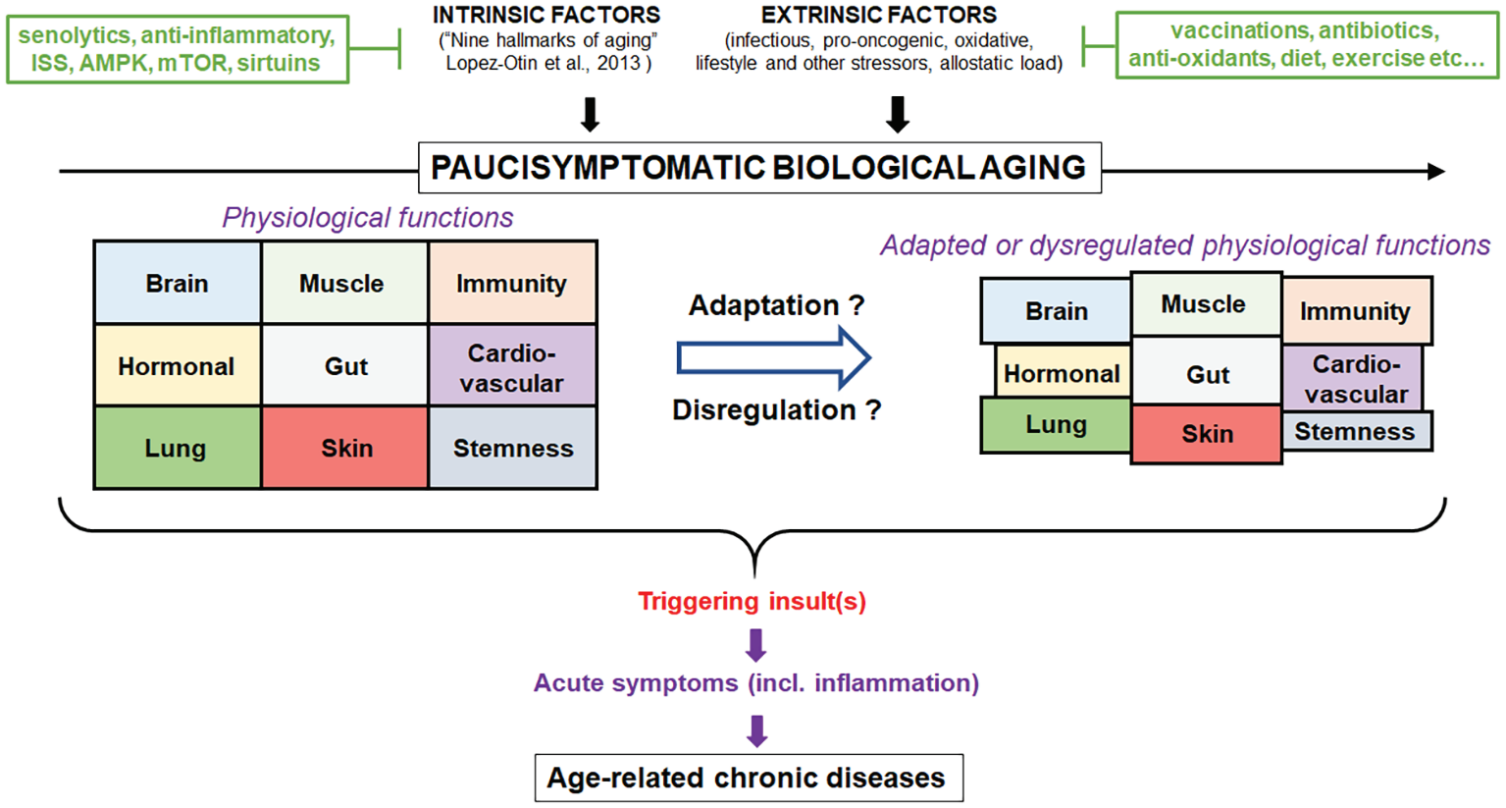
Conceptualization of the contribution of aging as a physiological process. Biological aging is a normal process that results from intrinsic (hallmarks of aging) and extrinsic factors (stress). In the absence of major insult during lifespan, individuals will experience very few symptoms (paucisymptomatic) related to change in health status despite the loss of physiological functions. However, upon certain triggering, uncontrolled inflammation may occur resulting in a persistent activation and disbalance of physiological functions leading to age-related diseases. Intervention strategies to modulate aging and reduce the susceptibility to diseases are represented. Aging can only be successfully modulated as long as it is considered a multifaceted process and not a disease in need of a specific cure. Ultimately, aging represents a time-dependent lifelong process, where under constant pressure the organism adapts or dysregulates which excludes the concept that aging is a disease.

Scientists should recognize at this stage that we know a lot but not enough yet to translate the scientific discoveries in the field of gerontology to interventions into the older subjects. However, a new approach is needed and should be oriented at a systemic conceptualization of the aging process and not at the fragmentation of its different components. Thus, better assessment of the biological aging against the chronological aging holds promises to be able (e.g., by significant biomarkers) to assess the physiological aging processes in their complexity and act on them specifically and jointly (Zhong et al., 2019). The concept that aging does not always lead to ARD, but that the same processes may lead to either ARD or successful aging in older persons depending on the homeodynamics, will also help to individualize the interventions (Franceschi et al., 2018). Furthermore, the recognition that not everything occurring in aging is detrimental will help to design purposeful interventions to reinforce what is necessary and combat what IS detrimental. Finally, the recognition of aging as a lifelong process and that chronic diseases start early in life would help to design interventions very early in life having consequences on ARD. This new concept would certainly help integrate the individual disease concept with aging-related dynamic dysregulation, the two being interconnected and intertwined. Depending on the preponderance of one or the other, the interventions may be targeted, respectively. So, we should move from the aging as a disease concept to the aging as an adaptation, which may result in ARD or successful functional healthspan (Figure 1).

## What can be Done to Face the Aging of the Society?

Besides direct intervention in the aging process (being the domain of anti-aging medicine), a healthy lifestyle should be advocated from the beginning of life, as an approach delaying both the occurrence of aging itself and that of ARDs (Saint Martin et al., 2017; Kaur et al., 2019) The first steps would be to implement healthy food, physical activity, and decreased stress (Bowen et al., 2019; Park and Lee, 2019). This seems to be wishful thinking; however, this approach would likely be more rewarding than non-specifically targeting aging. An increasing capacity to measure aging, imperfect though these methods may be, will help develop more specific lifestyle interventions for aspects of aging that are most relevant for health, and most urgent for a given individual.

This does not mean that research should not refine what are the biological pathways pertinent to aging. On the contrary, much should be invested in this research, but not with the undifferentiated aim that aging should be without disease. The aim should be to have an aging as late and as healthy as possible, contributing to the maintenance of the independent functionality of the aged subjects. This would join the aspirations of many elderly who know that aging without disease is almost impossible, but what is possible, and what all elderly aspire to, is remaining functional, independent, and not suffering from pain of any kind. The most important is not how long the elderly live, but how they should live the last years of their life. So, we should perhaps combine healthspan with functionspan, which could be the era of *functional healthspan*.

There should be several axes of approach to assure functional healthy aging for the elderly population, taking into account that these approaches may be different for each individual (Neubauer et al., 2017).

## Conclusion

Our modern societies face an unprecedented aging population increase. Science as well as the society should respond to this challenge. Science should unravel the complexity of the aging process and assure that interventions will lead to the maintenance of a health status permitting optimal functioning according to the wishes and priorities of individual persons. A healthy lifestyle to decrease the occurrence of chronic diseases and their accompanying functional burden is also a very important facet of possible interventions.

Aging is an irreversible, unstoppable, time-dependent process that is neither detrimental nor good but should be assessed in the individual context, which would permit an individualized (not “one size fits all”) intervention to adjust the process to optimize functioning in the aging body. Aging in this context should be considered as an adaptation of the organism as a function of chronic challenges and time; it is a necessary process but may be detrimental for responses to new challenges. The discovery of new processes and the integration of genetic/epigenetic/metabolic and environmental factors (including nutrition) by the virtue of systems biology approaches would nuance our “detrimental senescence” concept of aging.

Therefore, aging is not a disease, but a complex natural process. If aging was a disease, all elderly would be considered ill; but ultimately, if this was the common paradigm nobody would be anymore ill as this would become the norm (there is no disease universal for everybody). We can only modulate aging as long as we do not consider it as a disease. Treatment should be reserved for diseases, whatever their cause. This positive approach to aging would assure a functional healthspan in a personalized (individualized) way for each elderly subject, reducing the burden of the “silver tsunami.”

## Author Contributions

All authors contributed equally to the article by conceptualizing, discussing, and writing it. Furthermore, AL conceptualized the Figure 1.

### Conflict of Interest

AC is the founder and Chief Scientific Officer at Oken.

The remaining authors declare that the research was conducted in the absence of any commercial or financial relationships that could be construed as a potential conflict of interest.

The handling editor is currently organizing a Research Topic with AL, and confirms the absence of any other collaboration.
